# Supplementary material for: Promoting ordering degree of intermetallic fuel cell catalysts by low-melting-point metal doping
Source: Nat Commun. 2023 Sep 22;14:5896. doi: 10.1038/s41467-023-41590-2 (PMC10516855; doi:10.1038/s41467-023-41590-2)
Supplement: Supplementary file 1 — Supplementary Information [file 41467_2023_41590_MOESM1_ESM.pdf]

Supplementary Materials for

Promoting Ordering Degree of Intermetallic Fuel Cell Catalysts by Low-Melting-Point Metal Doping

Ru-Yang Shao,[1†] Xiao-Chu Xu,[2†] Zhen-Hua Zhou,[2] Wei-Jie Zeng,[2] Tian-Wei Song,[2] Peng Yin,[1] Ang Li,[2] Chang-Song Ma,[2] Lei Tong,[1,2] Yuan Kong,[1,3] Hai-Wei Liang[1,2]**

[1]Hefei National Research Center for Physical Sciences at the Microscale, University of Science and Technology of China, Hefei, 230026, China.

[2]Department of Chemistry, University of Science and Technology of China, Hefei, 230026, China.

[3]Department of Chemical Physics, University of Science and Technology of China, Hefei, 230026, China.

* Corresponding Author: Yuan Kong - kongyuan@ustc.edu.cn; Hai-Wei Liang - hwliang@ustc.edu.cn

† These authors contributed equally to this work.

This PDF file includes:

Supplementary Materials and Methods
Figs. S1 to S21
Tables S1 to S6

Supplementary Materials and Methods

Chemicals.

30 wt% Pt/C (TKK) was purchased commercially and used as received. Perchloric acid ($HClO_4$, 70%) and 5wt% Nafion solution were purchased from Sigma-Aldrich. Chloroplatinic acid ($H_2PtCl_6{\cdot}(H_2O)_6$), cobalt chloride hexahydrate ($CoCl_2{\cdot}6H_2O$, 99%), iron chloride anhydrous ($FeCl_3$, 99%), nickel chloride hexahydrate ($NiCl_2$, 99%), gallium chloride ($GaCl_3$, 99%), lead nitrate ($Pb(NO_3)_2$, 99%), antimonous chloride ($SbCl_3$, 99%), copper chloride dihydrate ($CuCl_2{\cdot}2H_2O$, 99%) and isopropanol (99.7%) were commercially available from Sinopharm Chemical Reagent Co. Ltd., China. All the chemicals were used as received without further purification. Deionized water (18.2 MΩ/cm) used in all experiments was prepared by passing through an ultra-pure purification system.

Theoretical Evaluation of Activity

The computational hydrogen electrode model was used for the expression of the chemical potentials of protons and electrons at any given pH and applied potential as in the ORR; The elementary steps by which the four-electron ORR occurs are believed to involve adsorbed OH, OO, OOH, and O species on the surface (*) according to the following:

$$* + O_2 \rightarrow OO^* \quad (1)$$

$$OO^* + H^+ + e^- \rightarrow OOH^* \quad (2)$$

$$OOH^* + H^+ + e^- \rightarrow H_2O + O^* \quad (3)$$

$$O^* + H^+ + e^- \rightarrow OH^* \quad (4)$$

$$OH^* + H^+ + e^- \rightarrow * + H_2O \quad (5)$$

Thus, the Gibbs free energy change for steps 1-5 can be expressed as:

$$\Delta G_1 = 4.92\ [eV] + G_{OO^*} - G_* \quad (6)$$

$$\Delta G_2 = G_{OOH^*} - G_{OO^*} - G_{H+}(pH) \quad (7)$$

$$\Delta G_3 = G_{H2O} + \Delta G_{O^*} - \Delta G_{OOH^*} - \Delta G_{H+}(pH) \quad (8)$$

$$\Delta G_4 = \Delta G_{OH^*} - \Delta G_{O^*} - \Delta G_{H+}(pH) \quad (9)$$

$$\Delta G_5 = \Delta G_{*} - \Delta G_{OH*} - \Delta G_{H+}(pH) \quad (10)$$

where $\Delta G_{H+}(pH) = - kBTln(10) \times pH$ is the free energy change at a nonzero pH value. Because the O_2 bond energy is difficult to determine by DFT calculations, the sum of $\Delta G_{1\text{-}5}$ was fixed to the experimental Gibbs free energy of -4.92 eV for forming two water molecules. The Gibbs free energy corrections of *OH, *O, and *OOH intermediates include zero-point energy (ZPE) and entropy corrections according to $\Delta G = \Delta E + ZPE - T\Delta S$.

The theoretical overpotential was then defined as:

$$\eta = \max \{\Delta G_1, 1.23V - |\Delta G_{2\text{-}5}/e^-|\} \quad (11)$$

Figs. S1 to S12

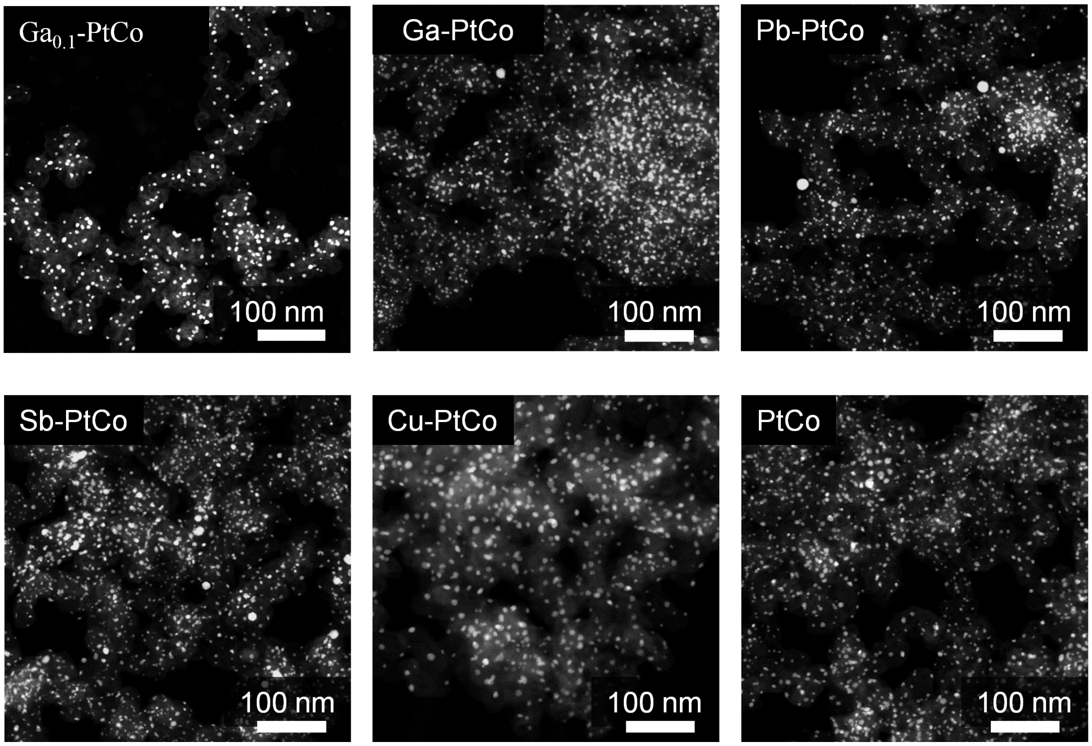

Fig. S1.
Low-magnification HAADF-STEM images of M-PtCo (M = Co, Cu, Sb, Pb, Ga).

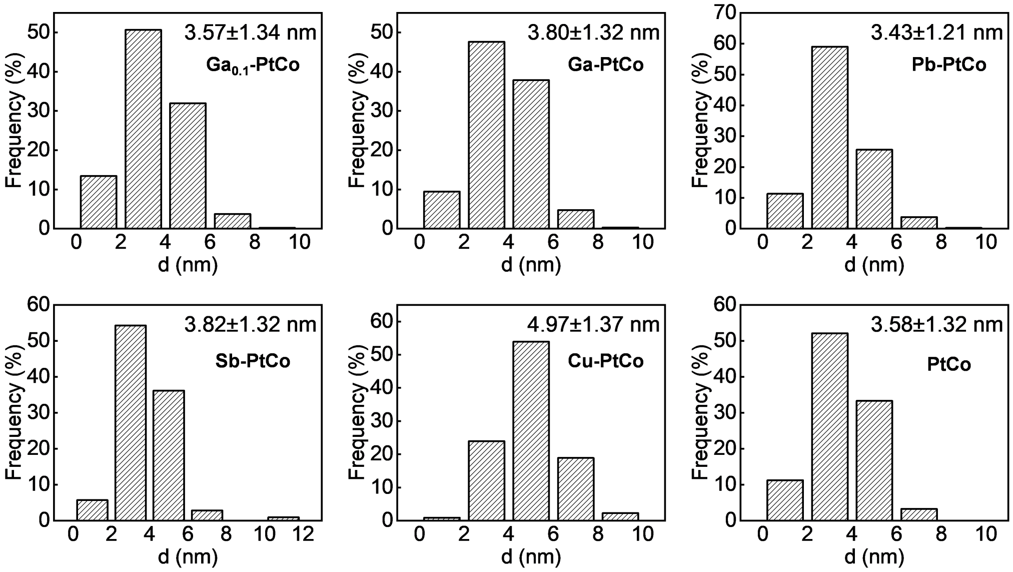

Fig. S2.

Statistics of particle size distribution of M-PtCo (M = Co, Cu, Sb, Pb, Ga).

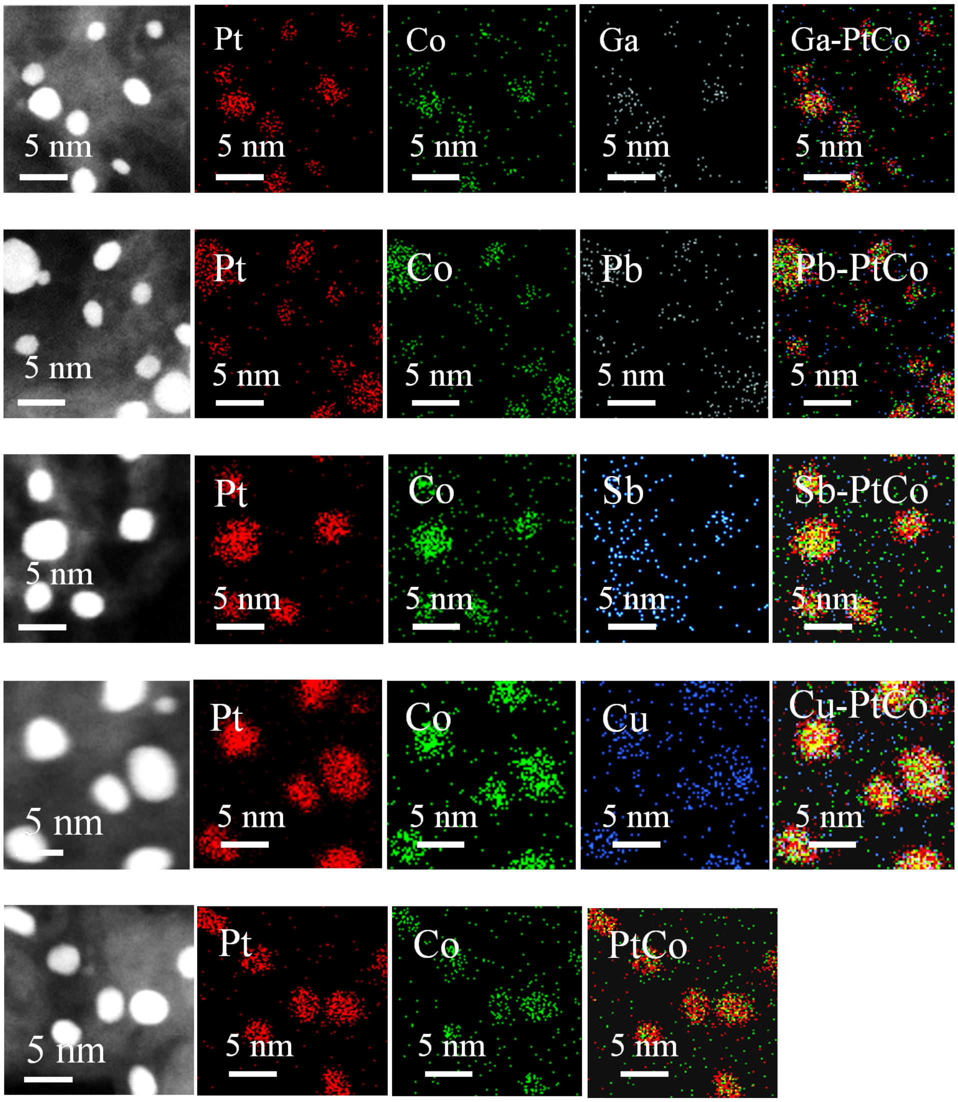

Fig. S3.
EDS elemental mappings of M-PtCo (M = Co, Cu, Sb, Pb, Ga) for TEM.

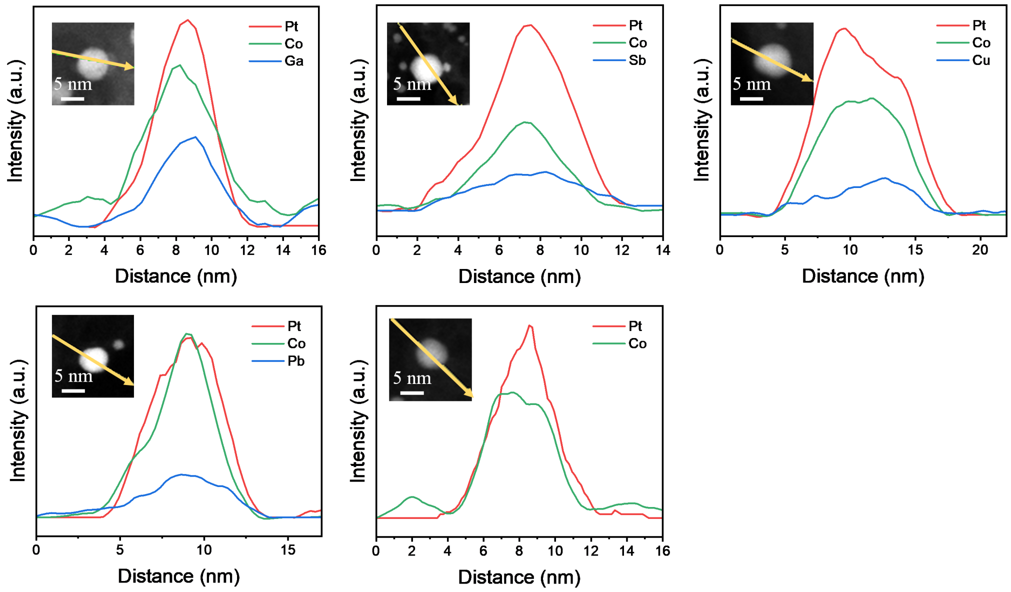

Fig. S4.

EDS line profile of M-PtCo (M = Co, Cu, Sb, Pb, Ga) for TEM along the insert yellow arrow.

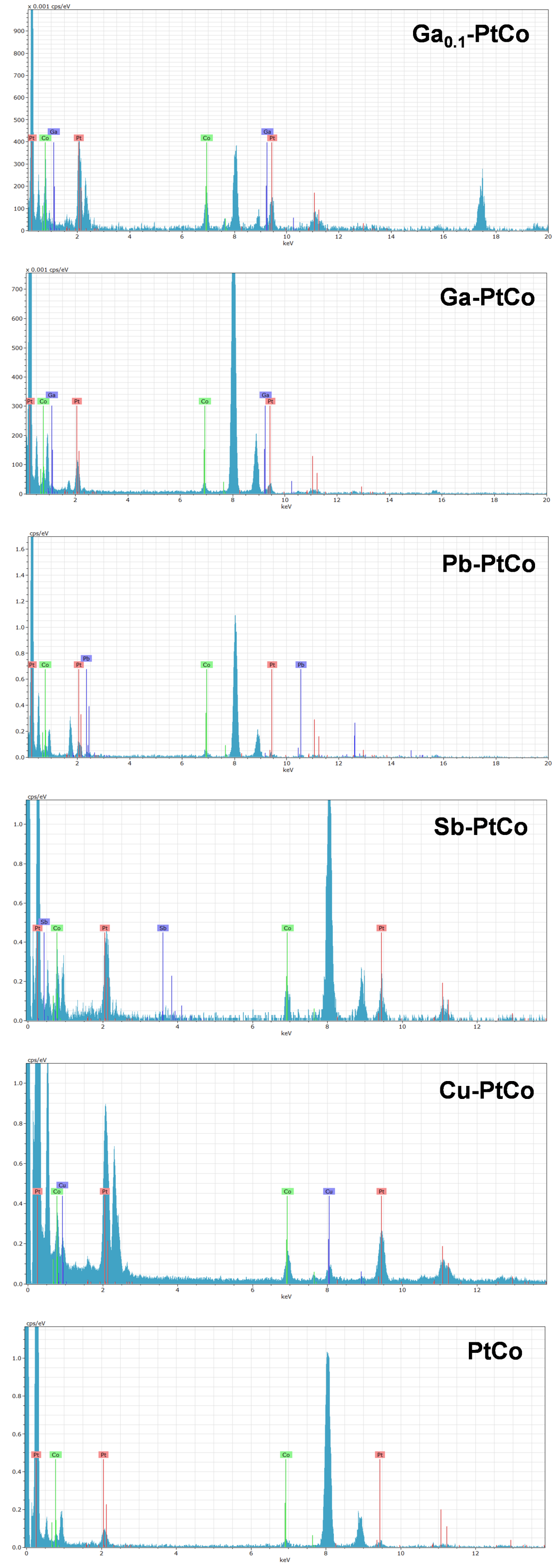

Fig. S5.
STEM-EDS spectra of M-PtCo (M= Co, Cu, Sb, Pb, Ga).

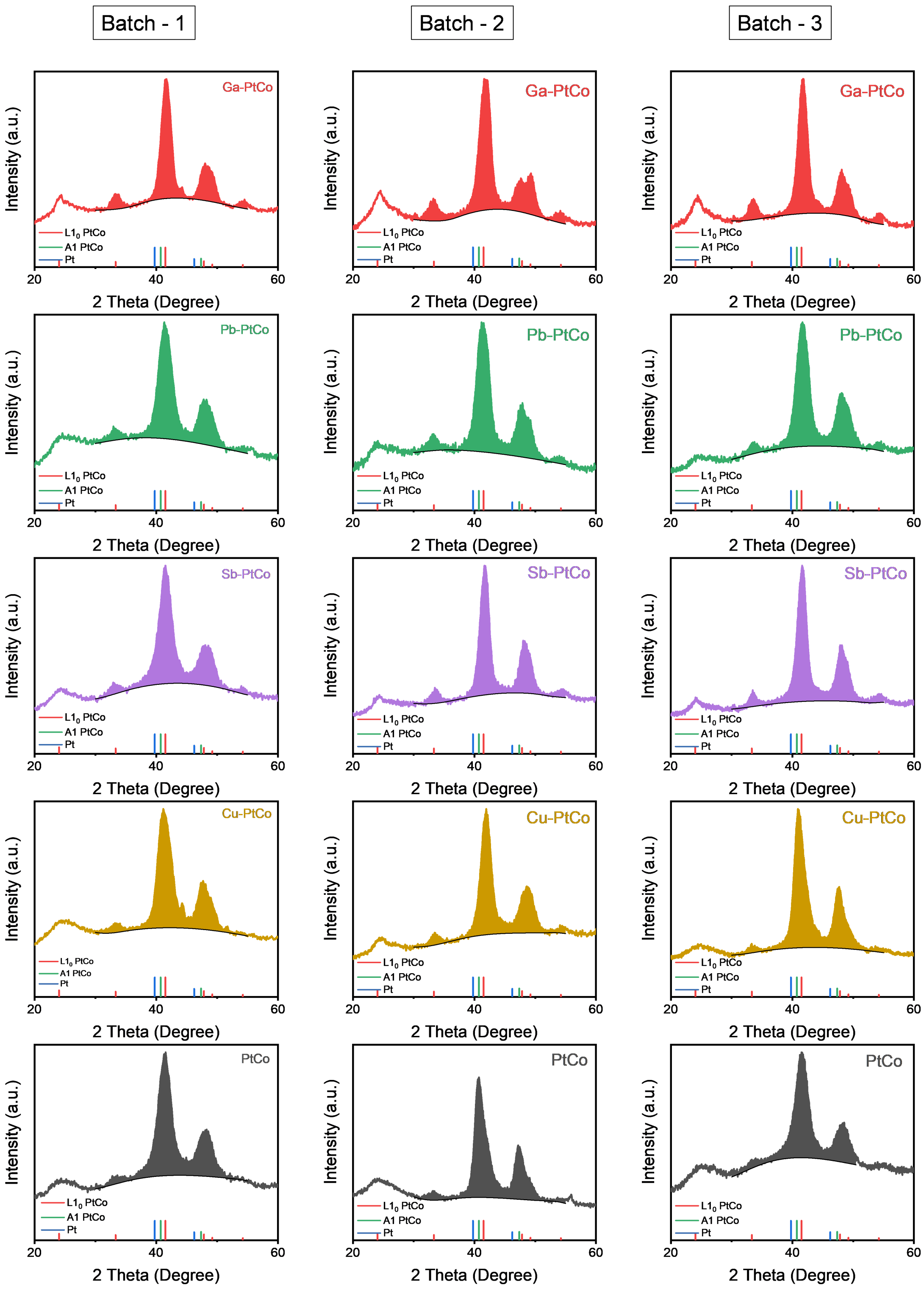

Fig. S6.

XRD patterns of M-PtCo (M = Co, Cu, Sb, Pb, Ga). All catalysts were synthesized and measured repeatedly by three times.

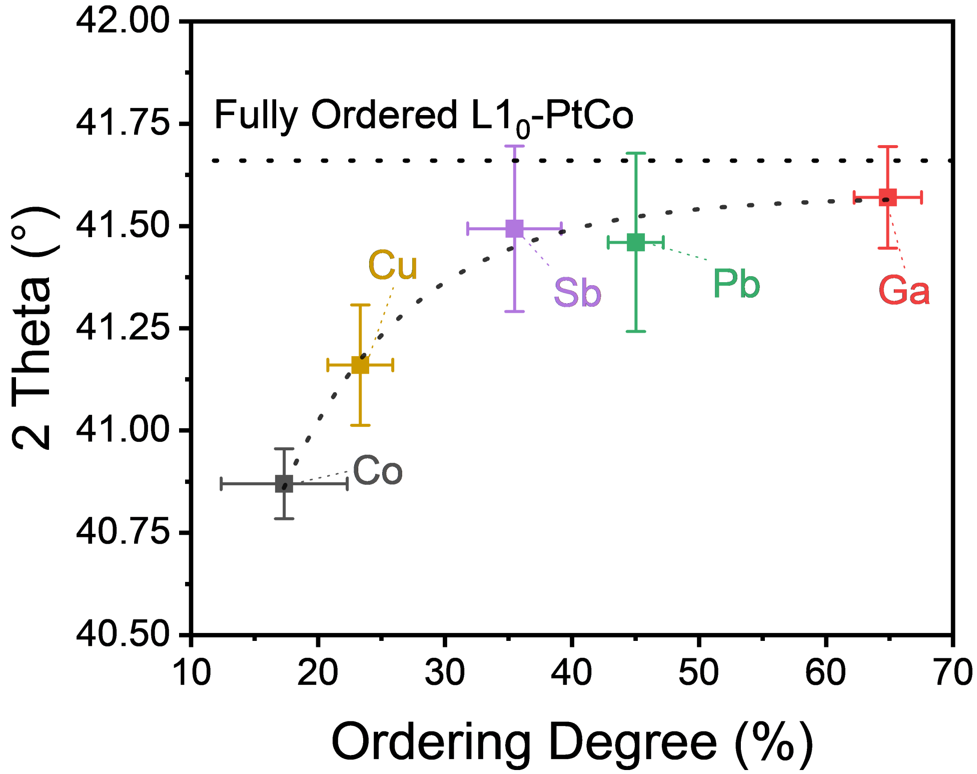

Fig. S7.

Dependence of ordering degree of the M-PtCo (M = Co, Cu, Sb, Pb, Ga) catalysts versus the position of (111) diffraction peak in XRD. The inserted horizontal dashed line represents the fully ordered $L1_0$-PtCo structure.

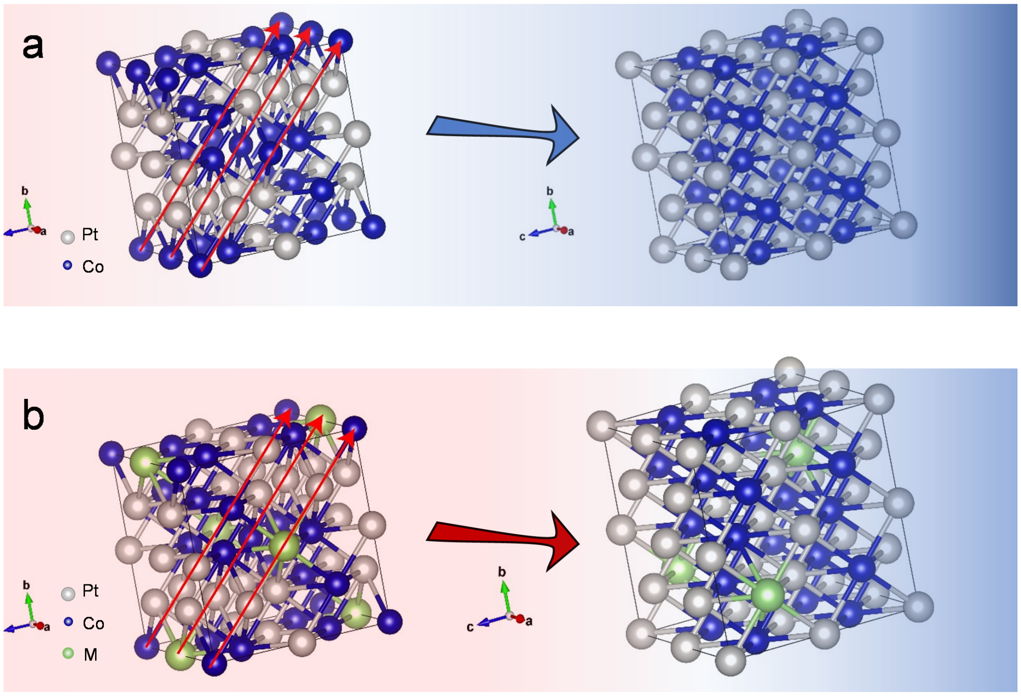

Fig. S8.

Atomic movement trajectory of disorderly converting into a linear diffusion model assumption. (a) Pure PtCo. (b) Low-melting-point metal doped M-PtCo. Atom colors: Pt (gray), Co (blue), and M (green, M= Co, Cu, Sb, Pb, Ga).

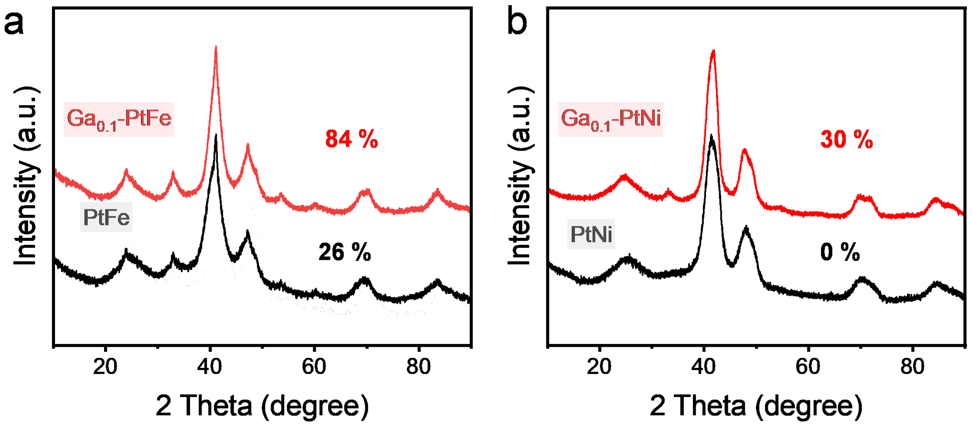

Fig. S9.
XRD patterns of Ga-doped and undoped $L1_0$ PtFe (A) and PtNi (B), respectively.

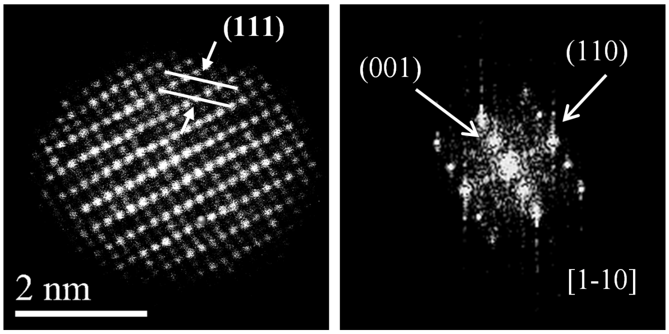

Fig. S10.

FFT patterns of the atomic-resolution HAADF-STEM image of $Ga_{0.1}$-PtCo.

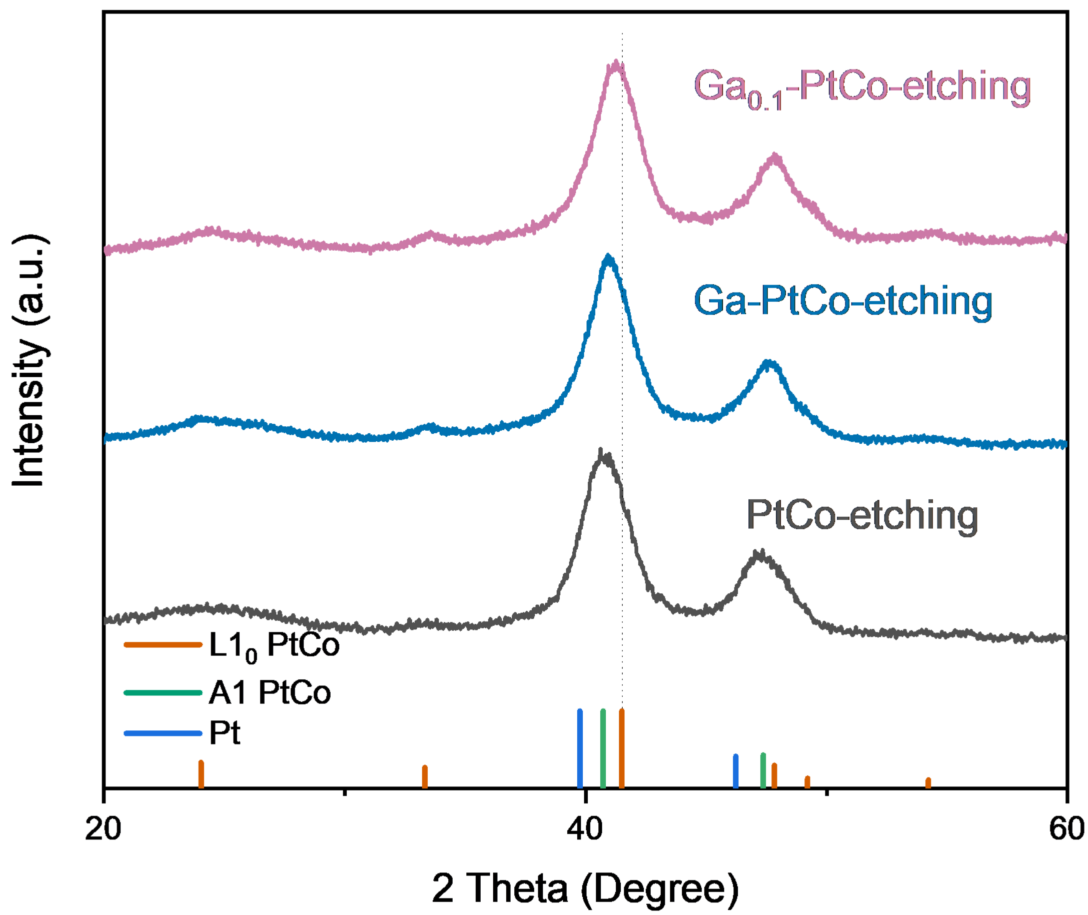

Fig. S11.

XRD patterns of $Ga_{0.1}$-PtCo, Ga-PtCo, and PtCo after acid leaching (0.1 M $HClO_4$, 60 °C, 1 h) and low-temperature annealing (400 ºC, 2 h, Ar/H_2) treatments.

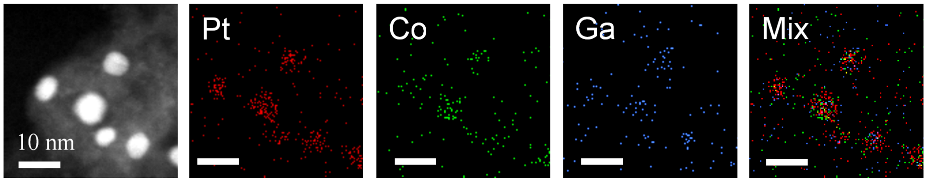

Fig. S12.

EDS elemental mappings of $Ga_{0.1}$-PtCo after acid leaching (0.1 M $HClO_4$, 60 °C, 1 h) and low-temperature annealing (400 ºC, 2 h, Ar/H_2) treatments. The insert scale bar is 10 nm.

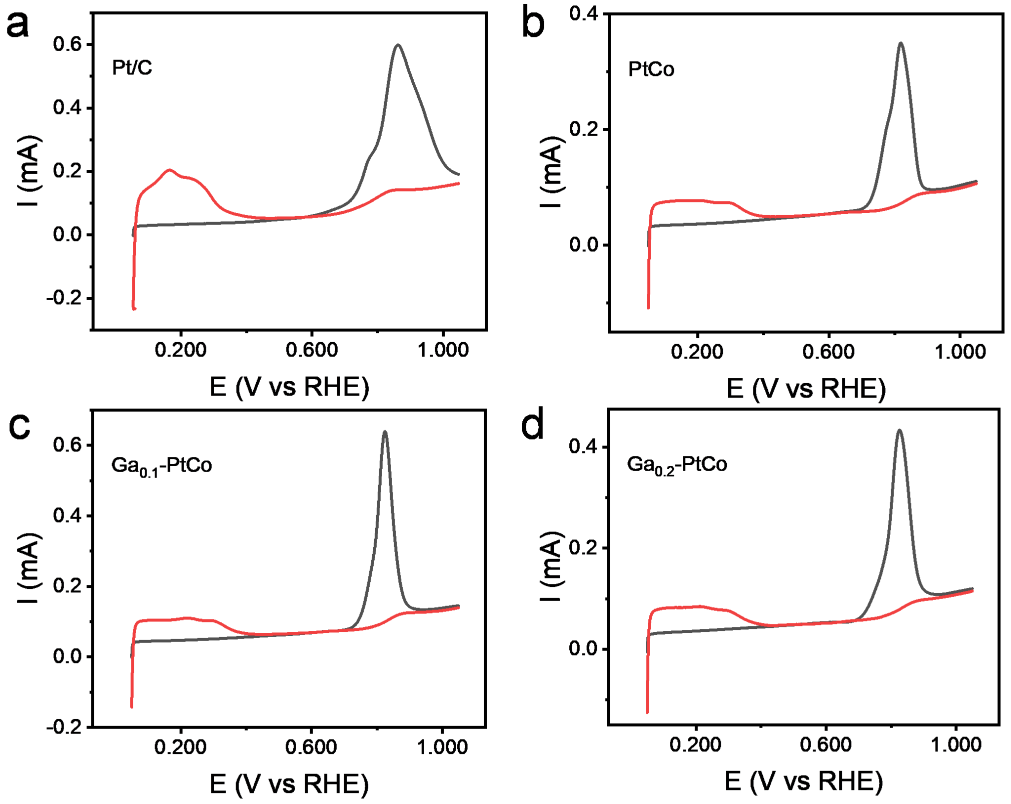

Fig. S13.

CO stripping curves of (a) commercial Pt/C, (b) PtCo, (c) $Ga_{0.1}$-PtCo, and (d) Ga-PtCo.

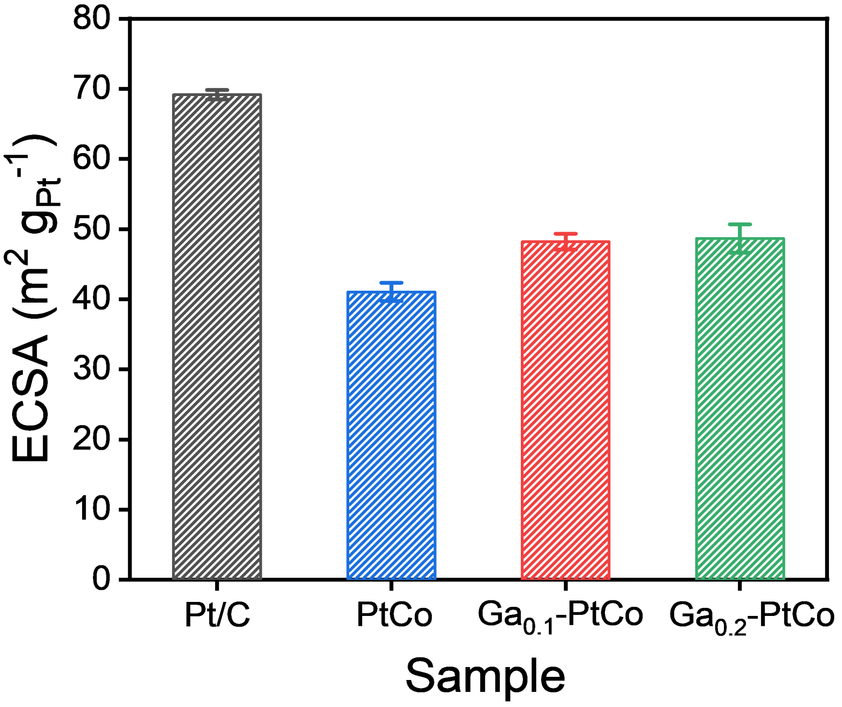

Fig. S14.

Electrochemical active areas of commercial Pt/C, PtCo, $Ga_{0.1}$-PtCo, and Ga-PtCo.

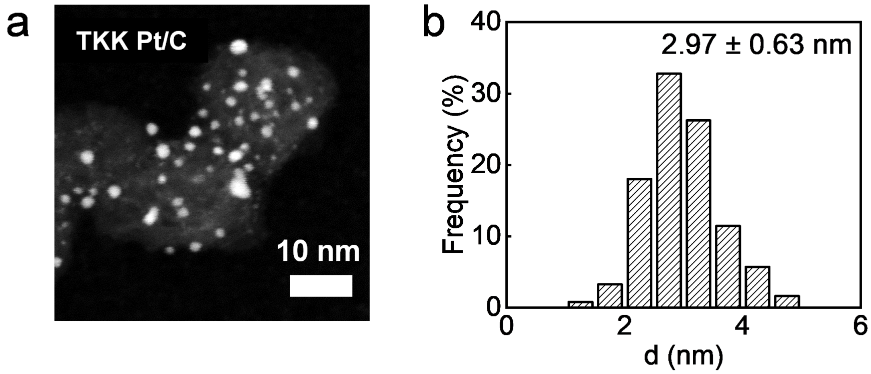

Fig. S15.
HADDF characterization of TKK 30wt% Pt/C. (a) HADDF-STEM image and (b) statistics of particle size distribution.

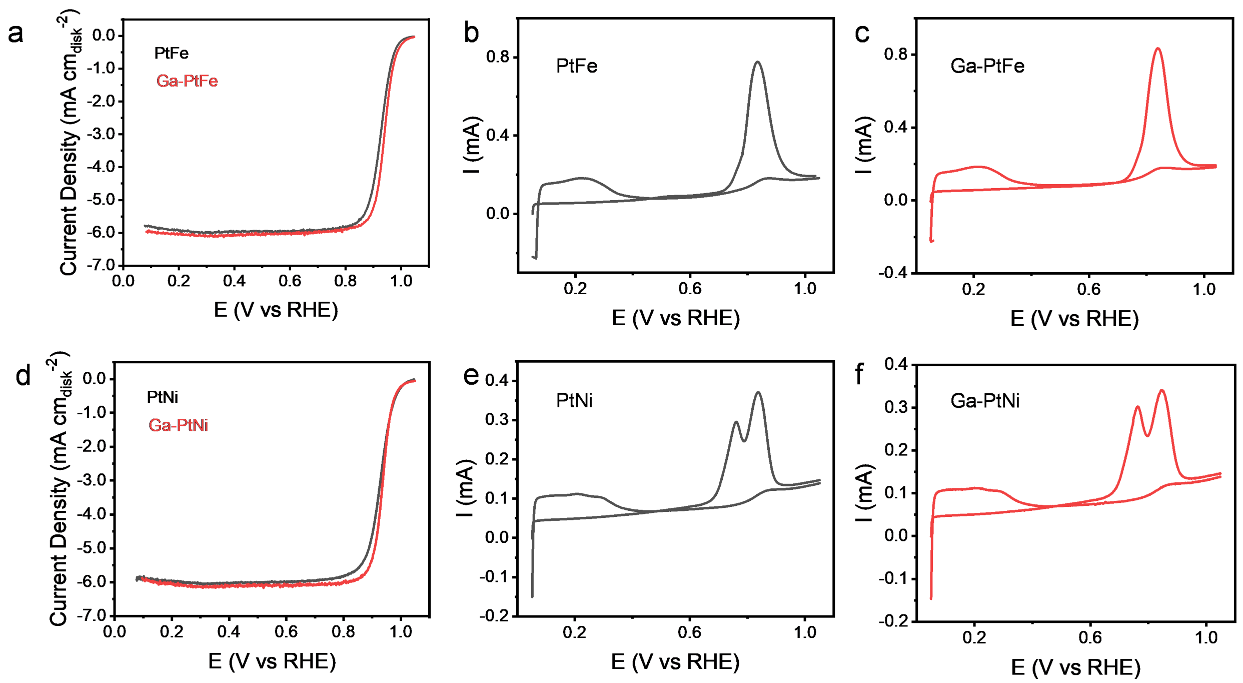

Fig. S16.

Electrocatalytic performance of Ga-doped and un-doped PtFe and PtNi. (a) ORR polarization curve of Ga-PtFe and PtFe in O_2-saturated 0.1 M $HClO_4$. (b, c) CO stripping curves of PtFe and Ga-PtFe, respectively. (d) ORR polarization curve of Ga-PtNi and PtNi in O_2-saturated 0.1 M $HClO_4$. (e, f) CO stripping curves of PtNi and Ga-PtNi, respectively.

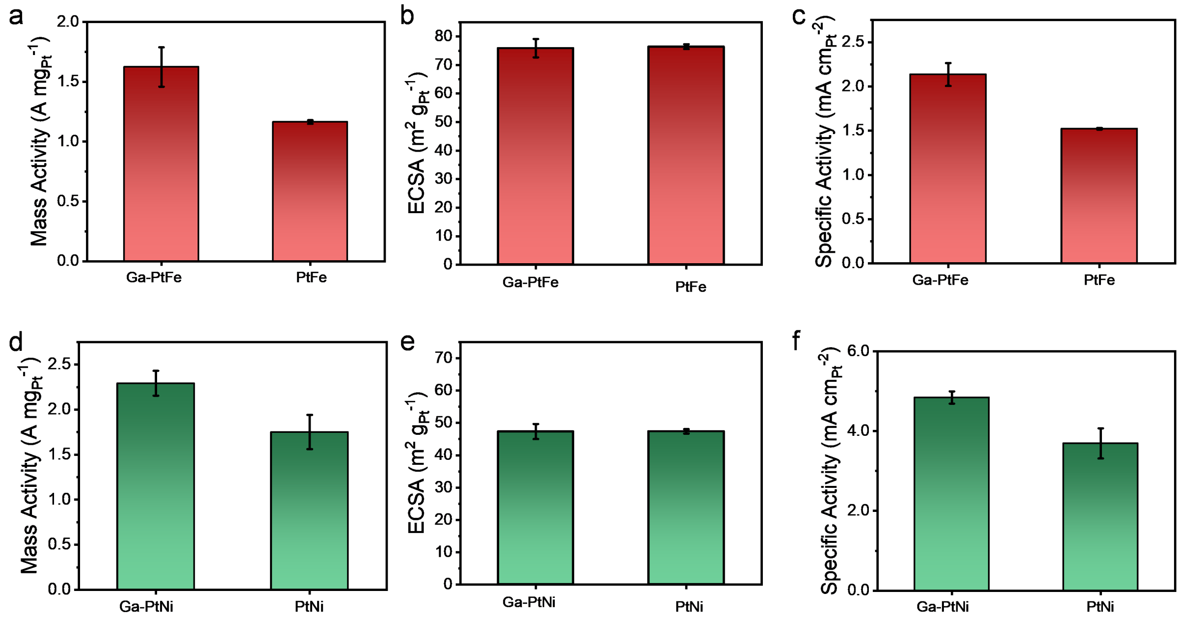

Fig. S17.

ORR activity of Ga-doped and undoped PtFe and PtNi at 0.9V. (a, d) Mass Activity, (b, e) Electrochemical Surface Area, and (c, f) Specific Activity.

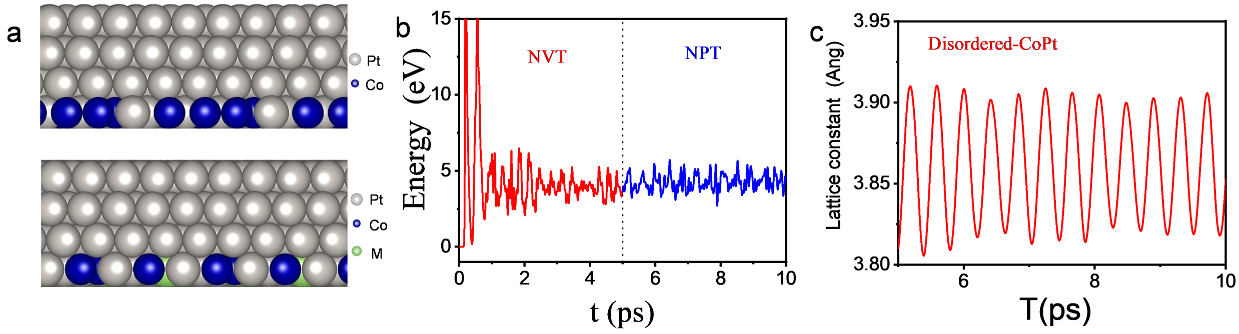

Fig. S18.

The simulated lattice parameter of A1 PtCo@Pt and L1_0 Ga-PtCo@Pt. (a) The atomic arrangement model for DFT calculation. Atom colors: Pt (gray), Co (blue), and Ga (green). (b) Function of energy and (c) lattice constant versus time during ab initio molecular dynamics (AIMD) simulation time on the corresponding Disordered–PtCo.

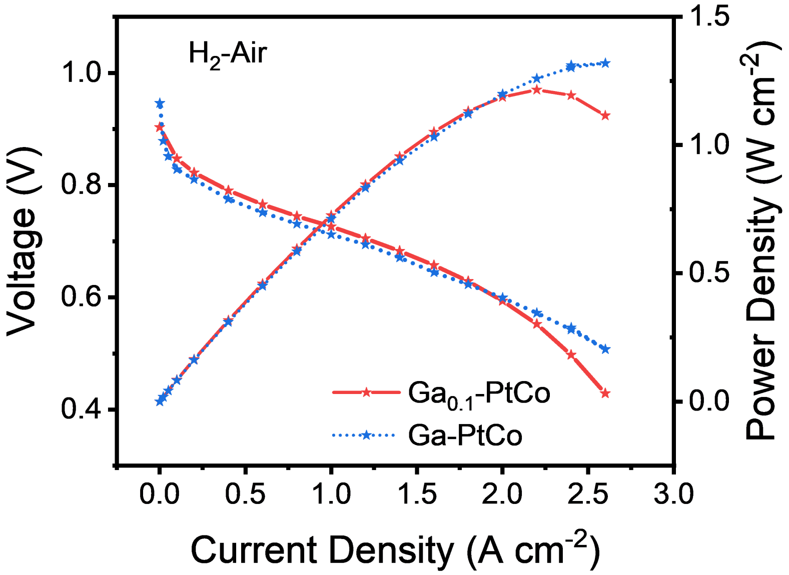

Fig. S19.

H_2-Air polarization curves and power densities of the $Ga_{0.1}$-PtCo and Ga-PtCo cathodes.

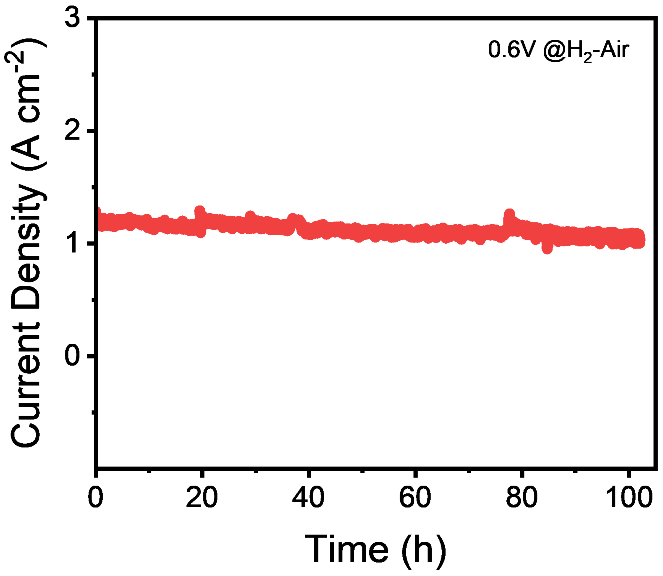

Fig. S20.

Current density as a function of time for $Ga_{0.1}$-PtCo at 0.6 V in H_2/air. Test conditions: 0.075 mg_{Pt} cm^{-2}, 80 °C, 100% relative humidity, 100 $kPa_{abs,\ outlet}$, H_2 and air flow rates were fixed at 0.2 and 0.5 liters min^{-1}, respectively).

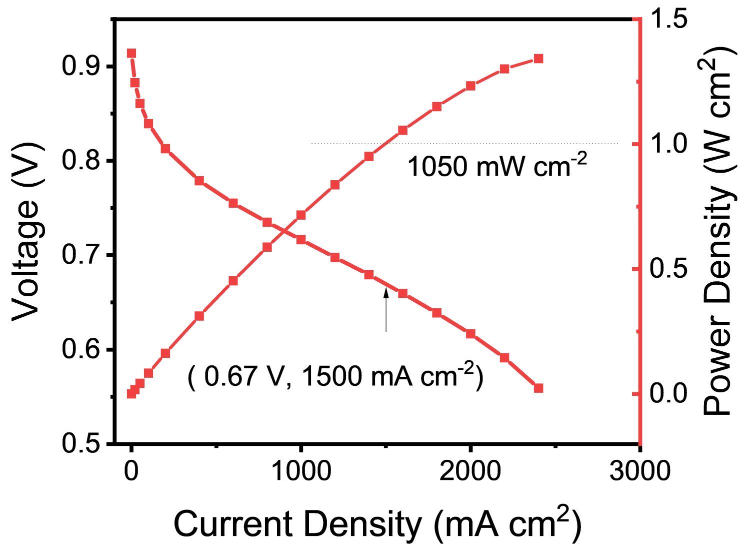

Fig. S21.

H_2-air fuel cell polarization curve and power density of the $PtCo_{0.9}Ga_{0.1}$ cathode at 94 °C. Test conditions: 94 °C, 65% relative humidity, 250 $kPa_{abs,\ outlet}$ H_2–air at high stoichiometries (H_2 and air flow rates were fixed at 0.5 and 2.0 liters min^{-1}, respectively). The rate power density at 0.67 V is marked by arrow.

Tables. S1 to S4

Table S1.
Structural information of the M-PtCo NPs catalysts.

	XRD Size (nm)	Number-average size (nm)	Volume-weighted size (nm)	(111) Peak position (º)
$Ga_{0.1}$-PtCo	4.01	3.57	4.87	41.74
Ga-PtCo	4.07	3.80	4.98	41.68
Pb-PtCo	3.74	3.43	4.66	41.27
Sb-PtCo	3.75	3.82	5.26	41.49
Cu-PtCo	4.21	4.97	6.01	41.11
PtCo	4.01	3.58	4.82	40.87
TKK Pt/C	-	2.97	3.36	-

Table S2.

Elemental composition of M-PtCo measured by ICP-AES and EDS mapping.

Sample	loading (wt%)	Pt (at%)		Co (at%)		M (at%)	
		EDS	ICP	EDS	ICP	EDS	ICP
Ga-PtCo	21.03	46.99	43.25	41.20	46.65	11.81	10.10
Pb-PtCo	20.76	42.79	46.29	47.62	47.88	9.59	5.83
Sb-PtCo	18.38	48.55	44.97	46.95	48.88	4.50	6.15
Cu-PtCo	17.59	45.34	46.04	43.76	45.18	10.91	8.78
PtCo	19.32	48.48	47.26	51.52	52.74	-	-
$Ga_{0.1}$-PtCo	20.62	53.12	45.74	42.54	50.10	4.34	4.16
Ga-PtCo etching	17.66	-	71.64	-	26.71	-	1.65
$Ga_{0.1}$-PtCo etching	18.08	61.75	62.02	37.07	36.84	1.18	1.14

Table S3.

Ordering degree of M-PtCo (M = Co, Cu, Sb, Pb, Ga) in Figure S6.

	Peak	Ga-PtCo	Pb-PtCo	Sb-PtCo	Cu-PtCo	PtCo
Batch-1	S(110)	0.2852	0.3053	0.2442	0.1644	0.1305
	S(111)	1.6462	2.2370	2.2189	2.3775	2.3793
	S(200)	0.6220	0.8793	0.8301	0.8869	1.0368
Batch-2	S(110)	0.3485	0.2554	0.1509	0.1169	0.1211
	S(111)	2.0972	2.3470	1.7277	1.8773	2.5133
	S(200)	0.8629	0.9701	0.7679	0.8997	1.1226
Batch-3	S(110)	0.3006	0.2215	0.1409	0.1008	0.0521
	S(111)	1.8926	1.8139	1.8675	2.2944	2.0276
	S(200)	0.6812	0.8072	0.8893	0.9551	0.7593
Average Ordering degree (%)		65.33 ± 2.67	47.06 ± 5.78	34.76 ± 8.04	22.39 ± 5.28	16.36 ± 5.52

Table S4.
ORR performance of Ga doped or undoped PtFe, PtCo, and PtNi at 0.9 and 0.95 V.

	MA@0.9V	SA@0.9V	MA@0.95V	SA@0.95V
	A mg_{Pt}^{-1}	mA cm_{Pt}^{-2}	A mg_{Pt}^{-1}	mA cm_{Pt}^{-2}
Pt/C	0.405 ± 0.053	0.586 ± 0.080	0.071 ± 0.003	0.103 ± 0.004
PtCo	1.450 ± 0.035	3.538 ± 0.199	0.147 ± 0.014	0.357 ± 0.037
$Ga_{0.1}$-PtCo	2.820 ± 0.226	5.847 ± 0.380	0.345 ± 0.010	0.717 ± 0.031
Ga-PtCo	2.303 ± 0.073	4.736 ± 0.161	0.209 ± 0.015	0.430 ± 0.023
PtFe	1.164 ± 0.015	1.522 ± 0.009	0.132 ± 0.006	0.172 ± 0.006
Ga-PtFe	1.624 ± 0.165	2.135 ± 0.130	0.170 ± 0.023	0.225 ± 0.037
PtNi	3.694 ± 0.379	3.694 ± 0.379	0.191 ± 0.003	0.402 ± 0.011
Ga-PtNi	4.843 ± 0.154	4.843 ± 0.154	0.200 ± 0.014	0.423 ± 0.029

Table S5.
Pressure drop of 7 flow channels flow field under H_2-Air test condition.

Temperature (ºC)	Air Flow Rate (L min^{-1})	Inlet Pressure (kPa_{abs})	Outlet Pressure (kPa_{abs})	Pressure Drop (kPa)
80	2	160	150	10
94	2	256	250	6

Table S6.

The protocol of hydrogen crossover and H_2-O_2 polarization curve for the determination of MA.

Voltage	Time	Temperature	Anode/Cathode RH	Anode flow rate	Cathode flow rate
Hydrogen Crossover					
0.3 V	2 min	80 ºC	100/100	H_2-0.5 L min^{-1}	N_2-0.05 L min^{-1}
0.4 V					
0.5 V					
0.6 V					
H_2-O_2 polarization					
0.775 V	4 min	80 ºC	100/100	H_2-0.2 L min^{-1}	O_2-0.2 L min^{-1}
0.800 V					
0.825 V					
0.850 V					
0.875 V					
0.900 V					
